# Integrated transcriptome and metabolome analysis unveil the response mechanism in wild rice (*Zizania latifolia* griseb.) against sheath rot infection

**DOI:** 10.3389/fgene.2023.1163464

**Published:** 2023-06-09

**Authors:** Limin Chen, Yamin Ma, Tianjun He, TingTing Chen, Yiming Pan, Dayun Zhou, Xiaowei Li, Yaobin Lu, Quancong Wu, Lailiang Wang

**Affiliations:** ^1^ Lishui Institute of Agriculture and Forestry Sciences, Lishui, Zhejiang, China; ^2^State Key Laboratory for Managing Biotic and Chemical Threats to Quality and Safety of Agro-products, Key Laboratory of Biotechnology in Plant Protection, Ministry of Agriculture and Rural Affairs, Institute of Plant Protection and Microbiology, Zhejiang Academy of Agricultural Sciences, Hangzhou, China; ^3^ Agricultural and Rural Bureau of Jinyun County, Jinyun, Zhejiang, China; ^4^ College of Plant Protection, Nanjing Agricultural University, Nanjing, China

**Keywords:** differentially accumulated metabolites, differentially expressed genes, *Fusarium asiaticum*, hypersensitive response, reactive oxygen species scavenging

## Abstract

Sheath rot disease (SRD) is one of the most devastating diseases of Manchurian wild rice (MWR) (*Zizania latifolia* Griseb). Pilot experiments in our laboratory have shown that an MWR cultivar “Zhejiao NO.7”exhibits signs of SRD tolerance. To explore the responses of Zhejiao No. 7 to SRD infection, we used a combined transcriptome and metabolome analysis approach. A total of 136 differentially accumulated metabolites (DAMs, 114 up- and 22 down-accumulated in FA compared to CK) were detected. These up-accumulated metabolites were enriched in tryptophan metabolism, amino acid biosynthesis, flavonoids, and phytohormone signaling. Transcriptome sequencing results showed the differential expression of 11,280 genes (DEGs, 5,933 up-, and 5,347 downregulated in FA compared to CK). The genes expressed in tryptophan metabolism, amino acid biosynthesis, phytohormone biosynthesis and signaling, and reactive oxygen species homeostasis confirmed the metabolite results. In addition, genes related to the cell wall, carbohydrate metabolism, and plant-pathogen interaction (especially hypersensitive response) showed changes in expression in response to SRD infection. These results provide a basis for understanding the response mechanisms in MWR to FA attack that can be used for breeding SRD-tolerant MWR.

## 1 Introduction

Sustainable food and nutrition security for a population of about 10 billion by 2050 is a top priority for researchers under unpredictable climatic conditions, reduced arable land, and increasing abiotic and biotic stresses (Hickey et al., 2019; Kumar et al., 2022). One crop that has been consumed as far back as 3,000 years ago in Zhou Dynasty is Chinese wild rice (*Zizania latifolia* Griseb) (Yan et al., 2018). Since its introduction to North America, it was once the staple food of the indigenous people and was commercialized in 1960 (Yu et al., 2020). It is one of the most important aquatic and economic vegetable crops s cultivated in southeast China (Guo et al., 2007). It is cultivated in more than ten provinces in China with nearly 100 thousand hectares (Zhang et al., 2022a). It grows well on sandy, loamy, or clay soils with either acid, neutral, or alkaline conditions under full Sun or partial shade. Sustainable production of Z. latifolia faces a number of diseases, including sheath rot (Terrell and Batra, 1982; Guo et al., 2015). Sheath rot has been reported to be caused by different fungal pathogens including *Sarocladium spp*. (*S. oryzae*)*, Fusarium spp*. (*Fusarium andiyazi, F. proliferatum, F. fujikuroi, F. verticillioides*), and *F. asiaticum* in different crops (Wulff et al., 2010; Bigirimana et al., 2015;)*. F. asiaticum* was first identified in barley in Japan (NRRL 13818) (O’Donnell et al., 2004). Bigirimana (2016) identified *F. asiaticum* as one of the pathogens associated with sheath rot in major rice-growing areas in Rwanda. The development of sheath rot lesions begins at the uppermost leaf sheaths surrounding young panicles. Early symptoms are observed as elongated to irregular lesions on the leaves with dark reddish-brown margins and brownish-grey color throughout (Jamaloddin et al., 2021).

The main virulence factors of sheath rot reported in the studies of Nandakumar et al. (2007); Ayyadurai et al. (2005) are the cell wall degrading enzymes and toxins cerulenin and helvolic acid. The disease affects chlorophyll biosynthesis, photosynthesis, and carbohydrate transport by scavenging magnesium ions (Farhat et al., 2016; Panda and Mishra, 2019). The pathogens mostly use toxins to suppress pathways (plant hormone signaling, phenylpropanoid and its related branches, etc.,) that confer resistance or alter essential developmental or physiological processes (Panda and Mishra, 2019; Peeters et al., 2020; Li et al., 2022). For example, Peeters et al. (2020) showed that *japonica* rice plants responded to highly toxin-producing *S. oryzae* and *P. fuscovaginae* strains by increasing abscisic acid (ABA), jasmonate (JA), and auxin (Aux) levels. In addition, lipids are the basic components of cell membranes, which serve as the first line of defense against pathogen invasion (Adigun et al., 2021). In order for plants to be attacked by fungal pathogens, the secreted toxins target plant lipid metabolism and induce an oxidative burst (Zhang et al., 2021).

To date, a variety of control strategies have been used, including cultural practices, fungicide application, balanced nutrients, growth regulators, organic amendments, and biological control using bacterial antagonists (Jamaloddin et al., 2021). In addition, the use of plant secondary metabolites has gained popularity in the control of plant pathogen/insect pest attacks (Kim et al., 2021; Li et al., 2022). For example, pyrroloquinoline quinone treatment induced rice resistance to sheath blight by regulating the jasmonic acid pathway. In addition, other anti-herbivore defense metabolites such as nicotine, caffeoyl putrescine, diterpene glycosides, and rutin, among others have been identified in previous studies (Baldwin, 1999; Keinänen et al., 2001; Steppuhn et al., 2004; Piasecka et al., 2015; Pusztahelyi et al., 2015; Adeniji et al., 2020; Castro-Moretti et al., 2020). A recent study by Kim et al. (2021) revealed that glutamic acid reshapes the plant microbiota to protect plants against pathogens in strawberry. Considering the fact that all high-yielding international rice cultivars are highly susceptible to sheath rot disease (Jamaloddin et al., 2021). We need to explore wild relatives that may contain resistance-related genes and metabolites that could be used in improving the high-yielding commercial cultivars for disease tolerance (Sakai and Itoh, 2010; Brar and Singh, 2011; Ehsan and Georgia, 2014; Brar and Khush, 2018). There are examples of successful introduction of introgression of tolerance-related genes/traits from wild to cultivated rice. For example, grassy stunt virus resistance from the AA-genome of *O. nivara* was transferred into the cultivated *O. sativa* (Khush et al., 1977). Similarly, several other genes/traits have been reported in wild relatives of rice (Brar and Singh, 2011). Regarding resistance to sheath rot pathogen, a number of wild accessions of rice have been reported to exhibit resistance (Vivekananthan et al., 2005). Thus, rice wild relatives are an opportunistic resource for tolerance-related genes/metabolites.

*Z. latifolia* Griseb (Manchurian wild rice, MWR), also known as water bamboo or Jiaobai, is an important wild rice species with economic, nutritional, and medicinal values (Zhai et al., 1996; Guo et al., 2007; Chu et al., 2018; Hou et al., 2020; Yu et al., 2021; Yan et al., 2022). It is one of the earliest important cereal crops in China and has been consumed as cereal for more than 3,000 years ago (Zhai et al., 1996). Z*. latifolia* has attracted much attention in recent years due to its long-term adaptation to environmental changes, resistance to abiotic and biotic stresses, nutritional and medicinal value, genetic variation, and useful genes (Guo et al., 2007; Guo et al., 2015; Chu et al., 2018; Mao et al., 2019; Yan et al., 2022). Our preliminary study showed that *Z. latifolia* cv “Zhejiao NO.7” (single-season type) shows resistance to sheath rot disease, which makes it an important resource for the identification of key metabolites and genes expressed in response to sheath rot infection.

Recent advances in transcriptome sequencing and metabolite profiling have opened new opportunities to identify biotic and abiotic stress tolerance/resistance mechanisms (Brar and Khush, 2018). Therefore, understanding the molecular mechanisms activated in response to sheath rot infection in wild rice (*Z. latifolia* Griseb.) could provide a basis for breeding sheath-rot-resistant cultivars. Here we report transcriptomic and metabolomic responses of MWR cv. Zhejiao NO.7 infected with *F. asiaticum* (FA).

## 2 Materials and methods

### 2.1 Pilot experiment

The plants showing visible symptoms of sheath rot were spotted in the field and were identified and validated by Professor Lailiang Wang (corresponding author). The symptoms included brown spots concentrated on the leaf sheaths. The symptomatic leaf sheath samples were collected from the field and the pathogen was identified as *Fusarium asiaticum* through isolation, purification, and morphological observation as previously reported (Wang et al., 2021). After the pathogen was identified as *F. asiaticum,* five conventional MWR cultivars (Meirenjiao, Zhejiao NO.7, Zhejiao NO.3, ShuiZhen NO.1, and Jinjiao NO.1) were tested for their resistance/susceptibility to *F. asiaticum* at different hours after infection, i.e., 0, 60, 72, 86, and 94 h. The preliminary data showed that Zhejiao NO. 7 showed signs of resistance [see (Ming’an et al., 2016)]. Therefore, we examined Zhejiao NO. 7 sheaths by transcriptome and metabolome analyses.

### 2.2 Plant material and growth conditions

Sterilized near-ground stems of *Z. latifolia* (Manchurian wild rice, MWR), cv. “Zhejiao NO.7,” grown in nutrient solution following instructions by (Yoshida et al., 1971) in plastic containers (60 cm × 40 cm x 35 cm), were transferred to a hydroponic incubator for 2 weeks to obtain fresh seedlings. The temperature, light, and relative humidity in the incubator were 28°C ± 2°C, 14 h light and 10 h dark, and 50%–60%, respectively. When the seedlings reached a height of 8–10 cm, they were transferred to a greenhouse, and allowed to grow for 30 days to the seven-leaf stage. Uniform seedlings without infection were selected and divided into two groups for further experiments.

The virulent strain FA12 of *F. asiaticum* was isolated from the infected plants of MWR from Hu town, Jinyun county, Lishui city, Zhejiang province, China. Cultures of FA12 strain were grown on potato dextrose agar (PDA) overlaid with a 9 cm cellophane membrane and incubated in the dark at 28°C ± 0.5°C for 4 days before being used for inoculation. For each treatment, 15 plants were selected for inoculation at a concentration of 1 × 10^7^ conidia mL^−1^. Briefly, a punch was used to make a small hole in the sheath at the same position, and then 10 µL of suspended spores were injected (hereafter FA). The control (CK) group of plants was injected with the same volume of distilled water as mock inoculation. The FA and CK plants were placed in the dark for 12 h and successfully inoculated plants were used for further analysis.

### 2.3 Metabolome analysis

#### 2.3.1 Sample preparation and UPLC-MS/MS analysis

Triplicate FS and CK samples were freeze-dried in a vacuum freeze-dryer (Scientz-100F) and ground using a mixer mill (MM 400, Retsch) with zirconia beads for 1.5 min at 30 Hz. The powder (100 mg) was dissolved in 1.2 mL 70% methanol solution, vortexed for 30 s after every 30 min 6 times, and placed at 4°C overnight. The next morning, the mixture was centrifuged at 12000 rpm for 10 min and the extracts were filtered (SCAA-104, 0.22 μm pore size; ANPEL, Shanghai, China) and analyzed in a UPLC-MS/MS system (UPLC, Shim-pack UFLC SHIMADZU CBM A system, https://www.shimadzu.com/; MS, QTRAP^®^ 4,500+ system, https://sciex.com/). The analytical conditions were as follows, UPLC: column, Waters ACQUITY UPLC HSS T3 C18 (1.8 µm, 2.1 mm*100 mm); column temperature, 40°C; flow rate, 0.4 mL/min; injection volume, 2 μL; solvent system, water (0.1% formic acid): acetonitrile (0.1% formic acid); gradient program, 95:5 V/V at 0 min, 5:95 V/V at 10.0 min, 5:95 V/V at 11.0 min, 95:5 V/V at 11.1 min, 95:5 V/V at 15.0 min.

The LIT and triple quadrupole (QQQ) scans were acquired on a triple quadrupole-linear ion trap mass spectrometer (Q TRAP), AB4500 Q TRAP UPLC/MS/MS System, equipped with an ESI Turbo Ion-Spray interface. The system operated in both positive and negative ion modes and was controlled by Analyst 1.6.3 software (AB Sciex). The ESI source operation parameters were as follows: ion source, turbo spray; source temperature 550°C; ion spray voltage (IS) 5500 V (positive ion mode)/−4500 V (negative ion mode); ion source gas I (GSI), gas II(GSII), curtain gas (CUR) were set to 50, 60, and 25.0 psi, respectively; the collision-activated dissociation (CAD) was high. Instrument tuning and mass calibration were performed with 10 and 100 μmol/L polypropylene glycol solutions in QQQ and LIT modes, respectively. QQQ scans were acquired as MRM experiments with collision gas (nitrogen) set to medium. DP and CE for individual MRM transitions were performed with further DP and CE optimization. A specific set of MRM transitions was monitored for each time according to the metabolites eluted within that time period.

#### 2.3.2 Metabolome data processing and analysis

The MS data were processed in Analyst 1.6.3 software. Based on the local metabolic database, the metabolites were analyzed quantitatively and qualitatively analyzed as previously reported (Chen et al., 2019). To compare the difference in the content of each metabolite in the samples among all the detected metabolites, the mass spectrum peaks detected in different samples were corrected for each metabolite according to the retention time and peak shape of the metabolites.

For statistical analysis, the missing values were assumed to be below the limits of detection, and these values were imputed with a minimum composite value, i.e., 9. The relative abundance of each metabolite was log-transformed and the individual metabolite abundances between FA and CK were compared by Dunnett’s test. Principal component analysis (PCA) was used for unsupervised pattern recognition. For hierarchical cluster analysis (HCA), the metabolite content data were normalized by unit variance scaling (UV), and the heatmap was generated in R using the *pheatmap* package. Pearson’s correlation coefficient (PCC) was calculated using the built-in cor function in R. In addition to PCA, orthogonal partial least squares-discriminant analysis (OPLS-DA) was used to maximize the metabolomic difference between the FA and CK samples. This was achieved by using MetaboAnalystR package *OPLSR.Anal* function in R as reported earlier (Thévenot et al., 2015). Based on the OPLS-DA results, the variable importance in projection (VIP) was used to screen the differentially accumulated metabolites (DAMs) between FA and CK. Finally, the DAMs were enriched in KEGG pathways (Arakawa et al., 2005) by Norminkoda Biotechnology Co., Ltd., (Wuhan, China).

### 2.4 Transcriptome analysis

#### 2.4.1 RNA extraction, library preparation, and sequencing

Triplicate FA and CK MWR samples stored at −80°C were used for total RNA extraction by using an RNA extraction kit (Name of the kit, XYZ, China). The purity, quantity, and integrity of the extracted RNAs were confirmed by using a NanoPhotometer spectrophotometer (IMPLEN, Los Angeles, CA, United States), Qubit RNA Assay Kit in Qubit 2.0 Flurometer (Life Technologies, Carlsbad, CA, United States), and RNA Nano 6000 Assay Kit of the Agilent Bioanalyzer 2,100 system (Agilent Technologies, Santa Clara, CA, United States), respectively. Sequencing libraries were prepared by using the NEB Next Ultra RNA Library Prep Kit as previously reported (Chen et al., 2019). Briefly, mRNAs were purified from total RNAs, and the first strand cDNAs were synthesized for each replicate of FA and CK. Next, the double-stranded (ds) cDNA was synthesized and purified according to the manufacturer’s protocol (NEB Next RNA Library Prep Kit). The dscDNAs were terminally repaired, A-tailed, and ligated with sequencing linkers, followed by fragment size selection. Finally, PCR enrichment was performed to obtain the final cDNA libraries. Preliminary quantification of the libraries was performed on a Qubit 3.0. Next, each library was diluted to 1 ng/uL and the insert size was determined by using an Agilent 2,100. Next, Q-PCR was then performed using a Bio-RAD KIT iQ SYBR GRN on a Bio-RAD CFX 96 fluorescence quantitative PCR instrument to accurately quantify the effective concentration of the library (>10 nM). The quality-qualified libraries were sequenced using an Illumina PE150 strategy.

#### 2.4.2 Bioinformatic analyses

The off-machine raw reads were processed to obtain clean reads; low-quality sequences and adapter contamination were removed. Next, the sequencing error rate and GC content were determined. The filtered sequences were compared to the reference genome using TopHat2 (Kim et al., 2013). For functional annotation, we used BLAST was used to compare sequences with the KEGG [36], GO [38], Nr (Deng et al., 2006), SwissProt (Apweiler, 2001), eggNOG/COG (Tatusov et al., 2000), KOG (Koonin et al., 2004), and Pfam (Finn et al., 2014) databases. Gene expression was measured as fragments per kilobase of transcript per million fragments mapped (FPKM), and the overall distribution of the gene expression was presented as a boxplot. The FPKM values were used for PCC, PCA, and HCA in R as described above. Differential expression analysis was performed using *DESeq2* package in R (Varet et al., 2016). The differentially expressed genes (DEGs) were screened based on log2 fold change (FC) (≥2) and adjusted *p*-value (padj) < 0.05. Functional-enrichment analysis including GO and KEGG was performed to identify which DEGs were significantly enriched in different metabolic pathways with Bonferroni-corrected *p*-value ≤0.05 compared with the whole-transcriptome background. GO functional enrichment and KEGG pathway analysis were carried out using Goatools (https://github.com/tanghaibao/Goatools) and KOBAS (http://kobas.cbi.pku.edu.cn/home.do).

### 2.5 Quantitative real-time PCR analyses

To validate the RNA-sequencing expression results, we randomly selected 12 DEGs and performed expression analysis by qRT-PCR. The primers were designed using the NCBI Primer Designing Tool (https://www.ncbi.nlm.nih.gov/tools/primer-blast/).

Total RNA was extracted by the CTAB method. The first strand of the reverse-transcribed cDNA was synthesized according to the specifications of the Monad first-strand cDNA Synthesis kits. Real-time fluorescence quantitative PCR was performed using the AB17500 quantitative PCR instrument. The rice *ubiquitin 5* gene (*OsUBQ5*) was selected as the reference gene in the study (Zhang et al., 2018). The list of primers used for the randomly selected DEGs and the housekeeping gene is shown in Supplementary Table S1. All data were obtained from three biological replicates and three technical replicates. The qRT-PCR analysis was performed by Norminkoda Biotechnology Co., Ltd. (Wuhan, China).

## 3 Results

### 3.1 Field observation of infected MWR plants and morphophysiological observation of *F. asiaticum*

**FIGURE 1:**
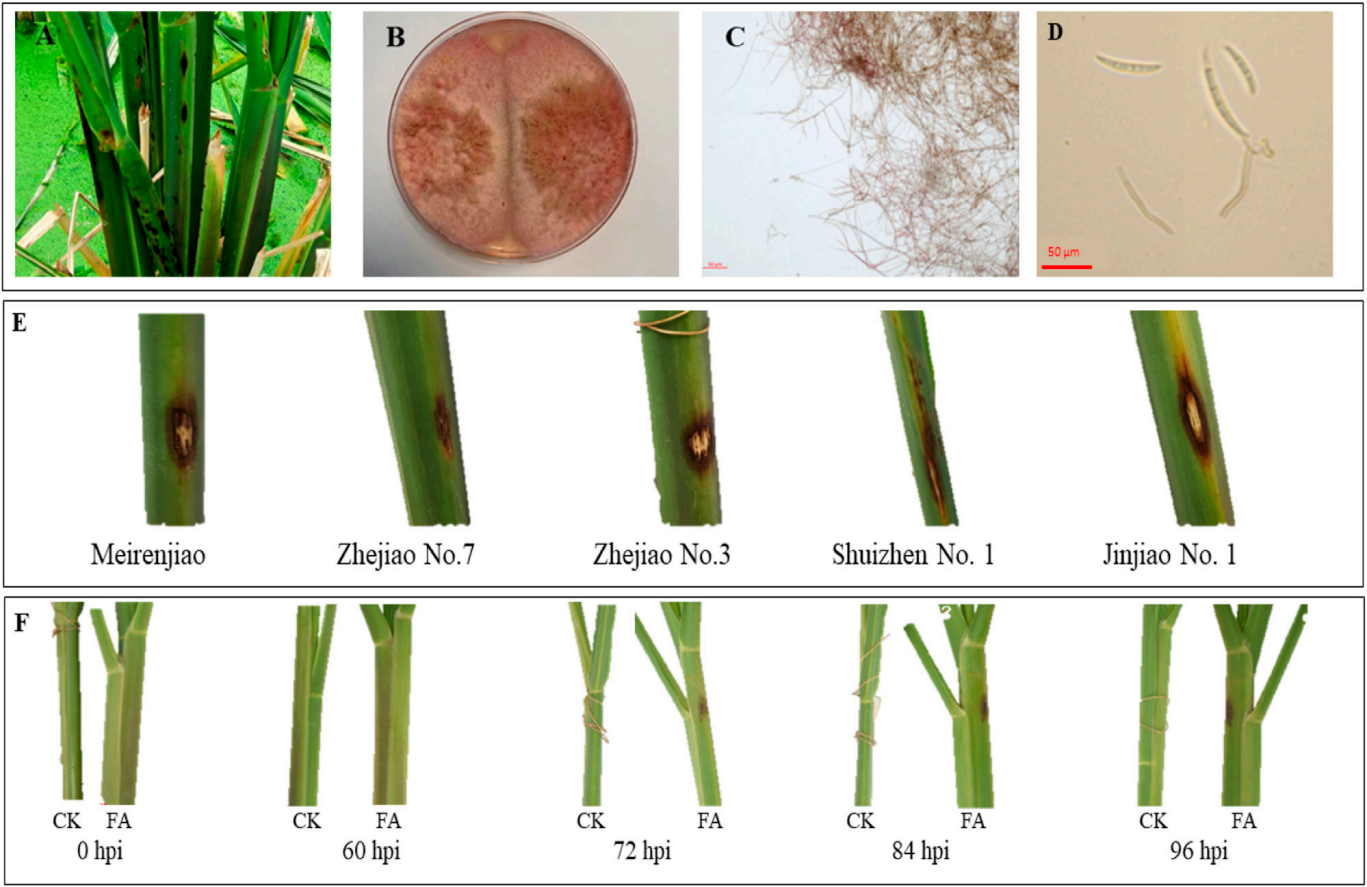
**(A)** Field symptoms of sheath rot of *Zizania latifolia*. **(B)** Growth status of PDA medium of *Fusarium asiaticum* pathogen. **(C)** Morphological characteristics of *F. asiaticum* hyphae. **(D)** Spore morphology of *F. asiaticum*. **(E)** The characteristics of 96-h spot of different varieties of *Z. latifolia* infected by *F. asiaticum*. **(F)** The characteristics of the disease spot of Zhejiao NO.7 infected by *F. asiaticum* at different times (hours post-inoculation, hpi).

Field observation of the sheath-rot-infected MWR showed that the disease spots were brown and mainly concentrated on the leaf sheath (Figure 1A). The pathogenic colony of *F. asiaticum* (FA) was pink, with obvious characteristics of hyphae and conidia (Figures 1B–D). Among the five cultivars tested in pilot experiments, i.e., Meirenjiao, Zhejiao No.7, Zhejiao No.3, ShuiZhen No.1, and Jinjiao No.1, Zhejiao NO.7 had a small disease spot indicating a better resistance compared to other four varieties (Figure 1E). The FA-infected Zhejiao No. 7 showed obvious pale brown spots (∼0.5 cm in diameter) on the leaf sheath. The lesion gradually increased in size with the passage of time and the color of the lesion gradually deepened. After 96 h of inoculation, the diameter of the lesion increased to 1–1.5 cm, and obvious necrotic lesions appeared on the leaf sheath. In contrast, there was no significant change in the leaf sheath of MWR treated with CK (Figure 1F).

### 3.2 Metabolomic changes in MWR in response to FA infection

**FIGURE 2:**
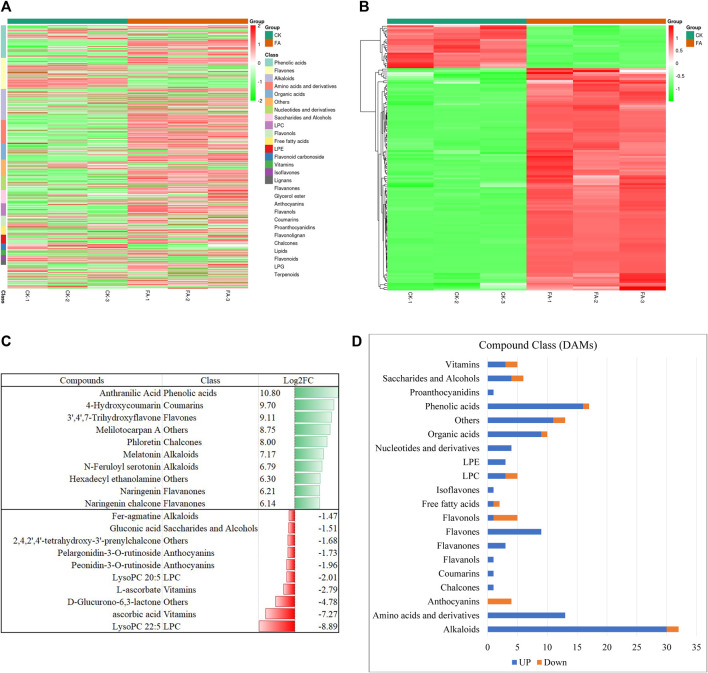
Summary of the metabolome profile of MWR before and after FA infection. **(A)** Heatmap of the relative intensities of the detected metabolites in CK and FA. **(B)** Heatmap of the relative intensities of the differentially accumulated metabolites in CK vs. FA. **(C)** Graphical representation of the top-10 up- and down accumulated metabolites in FA compared to CK. **(D)** Classification and regulation of the differentially accumulated metabolites in CK vs. FA. MWR = Manchurian wild rice, FA = MWR infected with *Fusarium asiaticum*, CK = control (no *F. asiaticum* infection). The numbers with CK and FA indicate replicates. The data in panels **(C,D)** are the mean of three replicates.

The UPLC-MS/MS-based global metabolome analysis of the FA and CK resulted in the detection of 796 metabolites belonging to 25 compound classes (Figure 2A). The highest number of detected metabolites were classified as phenolic acids (99), followed by flavones (94), alkaloids (93), amino acids and derivatives (70), and organic acids (50). PCA revealed a distinct grouping of the CK and FA replicates (Supplementary Figure S1A). Similarly, the cluster analysis showed the grouping of the replicates within the treatment (Supplementary Figure S1B). The PCC analysis showed that higher values of r^2^, i.e., (0.8–1.0) (Supplementary Figure S1C). The HCA, PCA, and PCC indicate that the sampling was reliable. Among the detected metabolites, 136 were DAMs; 114 and 22 were up- and downregulated in FA as compared to CK, respectively (Figure 2B). The highest number of DAMs was classified as alkaloids (23.5%), followed by phenolic acids (12.5%), amino acids and derivatives (9.55%), and organic acids (7.35%), etc. Among the compounds that were exclusively accumulated in FA include anthranilic acid, 4-hydroxycoumarin, 3′,4′,7′-trihydroxyflavone, melilotocarpan A, and phloretin. Furthermore, we also observed an increased accumulation of melatonin, N-feruloyl serotonin, hexadecyl ethanolamine, naringenin, and naringenin chalcone. In contrast, the metabolites whose levels were decreased in the infected MWR include ascorbic acid, D-glucurono-6-,3-lactone, L-ascorbate, peonidin-3-O-rutinoside, pelargonidin-3-O-rutinoside, 2,4,2′,4′-tetrahydroxy-3′-prenylchalcone, gluconic acid, feragmatine, peonidin-3-O-glucoside, and L-gulonic-γ-lactone (Supplementary Table S2; Figure 2C). From a compound class perspective, it was observed that all compound classes showed up-accumulation of all or the maximum number of metabolites in FA as compared to CK except anthocyanins (all down-accumulated) and flavonols (four out of five compounds were down-accumulated) (Supplementary Table S2; Figure 2D). These metabolites were significantly enriched in tryptophan metabolism, phenylalanine, tyrosine, and tryptophan biosynthesis, isoflavonoid biosynthesis, flavonoid biosynthesis, indole alkaloid biosynthesis, biosynthesis of amino acids, and anthocyanin biosynthesis (Supplementary Figure S2).

#### 3.2.1 Metabolomic changes in tryptophan biosynthesis/metabolism and related pathways

Based on KEGG pathway enrichment analysis, we specifically checked the changes in metabolites enriched in the tryptophan metabolism and related pathways. Interestingly, 13 DAMs were enriched in the tryptophan metabolism pathway. Compound accumulation indicated that anthranilate, indole, and tryptophan (enriched in phenylalanine, tyrosine, and tryptophan biosynthesis pathway) had higher accumulation in FA than CK. This higher indole and tryptophan accumulation, together with the increased accumulation of downstream metabolites leading to melatonin biosynthesis and indole acetate biosynthesis, is interesting because both of the compounds are known to play important roles in plant-pathogen interaction and disease resistance (Table 1; Supplementary Table S2) (Fu and Wang, 2011; Zeng et al., 2022).

**TABLE 1:** Differential accumulation of tryptophan, phenylpropanoid, and flavonoid biosynthesis-related metabolites in MWR after infection with FA.

Compounds	CK	FA	VIP	Foldchange	Log2FC
Tryptophan biosynthesis/metabolism					
Melatonin	11850	1706667	1.18	144.02	7.17
N-Feruloyl serotonin	10043	1113333	1.18	110.85	6.79
Tryptamine	332000	19700000	1.18	59.34	5.89
Nb-Trans-p-Coumaroylserotonin Glucoside	32133	232000	1.16	7.22	2.85
Serotonin	92600	501000	1.18	5.41	2.44
Methoxyindoleacetic acid	888000	3826667	1.18	4.31	2.11
Indole	189000	569000	1.18	3.01	1.59
3-Indoleacetonitrile	277000	787333	1.18	2.84	1.51
N-Acetyl-5-hydroxytryptamine	630667	1316667	1.17	2.09	1.06
L-Tryptophan	926667	3956667	1.18	4.27	2.09
Acetyltryptophan	2140000	6213333	1.18	2.90	1.54
N-Hydroxy tryptamine	39833	214333	1.17	5.38	2.43
Anthranilic Acid	100	178667	1.18	1786.67	10.80
Anthranilate O-hexosyl-O-hexoside	1320000	2876667	1.09	2.18	1.12
Phenylpropanoid biosynthesis					
p-Coumaryl alcohol	131333	377000	1.18	2.87	1.52
Cinnamic acid	5,710	14100	1.17	2.47	1.30
Coniferyl alcohol	346333	826333	1.18	2.39	1.25
Sinapic acid	1123333	2333333	1.17	2.08	1.05
Flavonoid biosynthesis					
Phloretin	100	25667	1.18	256.67	8.00
Pinobanksin	89400	6006667	1.18	67.19	6.07
Naringenin	74367	5516667	1.18	74.18	6.21
Naringenin chalcone	95633	6736667	1.18	70.44	6.14
Butin	59700	2356667	1.18	39.48	5.30
Galangin	50300	1870000	1.18	37.18	5.22
Apigenin	97500	2420000	1.18	24.82	4.63
Genistein (4′,5,7-Trihydroxyisoflavone)	93767	2473333	1.18	26.38	4.72

#### 3.2.2 Metabolomic changes in phenylpropanoid, flavonoid, and related pathways

Since DAMs were significantly enriched in phenylpropanoid and downstream flavonoid biosynthesis and related pathways, therefore, we specifically looked for changes in metabolite accumulation. In the case of the phenylpropanoid biosynthesis pathway, we observed the up-accumulation of phenolic acids (or intermediates) i.e., cinnamic acid, sinapic acid, p-coumaryl alcohol, and coniferyl-alcohol. These observations may suggest that MWR may activate cell wall changes, such as lignin modification by increasing phenolic acid accumulation under FA infection (Table 1; Supplementary Table S2). Furthermore, we observed the increased accumulation of indole-3-acetic acid (IAA) and abscisic acid (ABA) in FA compared to CK, suggesting the essential role of these hormones in MWR when infected with FA.

Similarly, the increased accumulation of flavonoids/flavanols/flavanones/flavones (phloretin, pinobanksin, galangin, butin, naringenin chalcone, naringenin, and apigenin) and isoflavonoid (genistein) (Table 1; Supplementary Table S3) may indicate that MWR activates the biosynthesis of these secondary metabolites to cope with FA infection similar to other plants such as chickpea (Kumar et al., 2015).

In addition to the pathways mentioned above, the increased accumulation of amino acids and derivatives, particularly, N-acetyl-L-glutamic acid, L-serine, L-histidine, and L-asparagine in FA suggests that amino acid metabolic pathways are an integral part of the MWR immune response to FA (Zeier, 2013). On the other hand, the increased accumulation of L-tartrate (enriched in glyoxylate and dicarboxylate metabolism) (Burbidge et al., 2021) and nicotinamide and nicotinate (enriched in nicotinate and nicotinamide metabolism pathway) (Miwa et al., 2017; Hong et al., 2020) may indicate increased antioxidant activity in FA plants compared to CK (Supplementary Table S3).

These observations suggest that MWR responds to FA infection by increasing the biosynthesis of major compound classes such as alkaloids, amino acids and derivatives, flavones, flavanones, nucleotides and derivatives, organic acids, phenolic acids, and saccharides and alcohols.

### 3.3 Comparative transcriptome of FA and CK MWR

**FIGURE 3:**
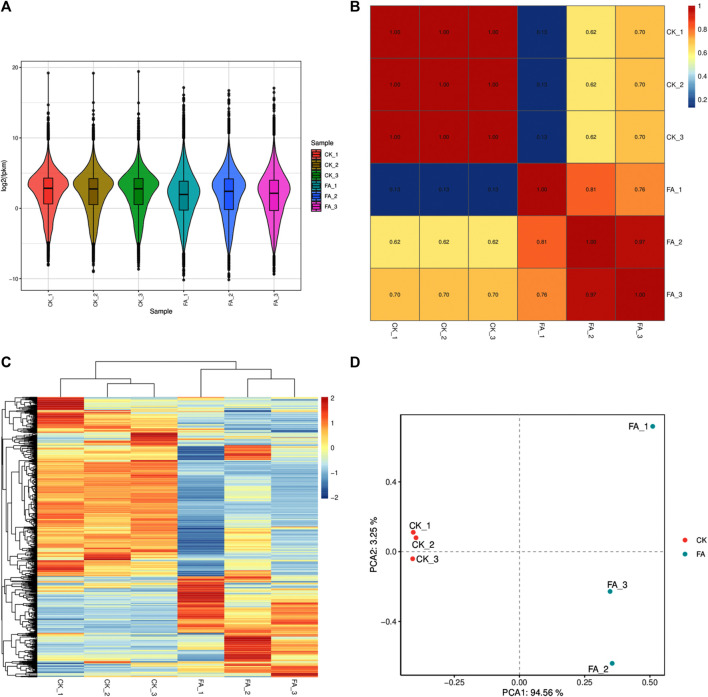
Comparative transcriptome analysis of CK vs. FA MWR. **(A)** Overall expression of the differentially expressed genes in CK and FA replicates. **(B)** Pearson’s Correlation Coefficient analysis, **(C)** hierarchical cluster analysis and heatmap, and **(D)** principal component analysis of the differentially expressed genes in CK and FA. MWR = Manchurian wild rice, FA = MWR infected with *Fusarium asiaticum*, CK = control (no *F. asiaticum* infection). The numbers with CK and FA indicate replicates.

The sequencing of six libraries resulted in an average of 75,726,899 raw reads. On average, 75,603,618 reads were obtained per library after removing adapters and low-quality reads, where bases with a quality value Q ≤ 10 accounted for more than 50% of the total reads. The GC content, >Q20%, and >Q30% were 55.22%, 98.003%, and 93.88%, respectively (Supplementary Table S3). Gene expression was calculated as FPKM, with the total FPKM of the FA replicates being lower than that of CK (Figure 3A). The PCC between the replicates of each treatment was high (∼1 for CK and ∼0.89 for FA) (Figure 3B), while the PCA and HCA analyses also indicated that treatment replicates were grouped together (Figures 3C, D). These results confirm that the sampling was reliable, as observed in the metabolomic analysis.

#### 3.3.1 Differential gene expression in CK vs FA

**FIGURE 4:**
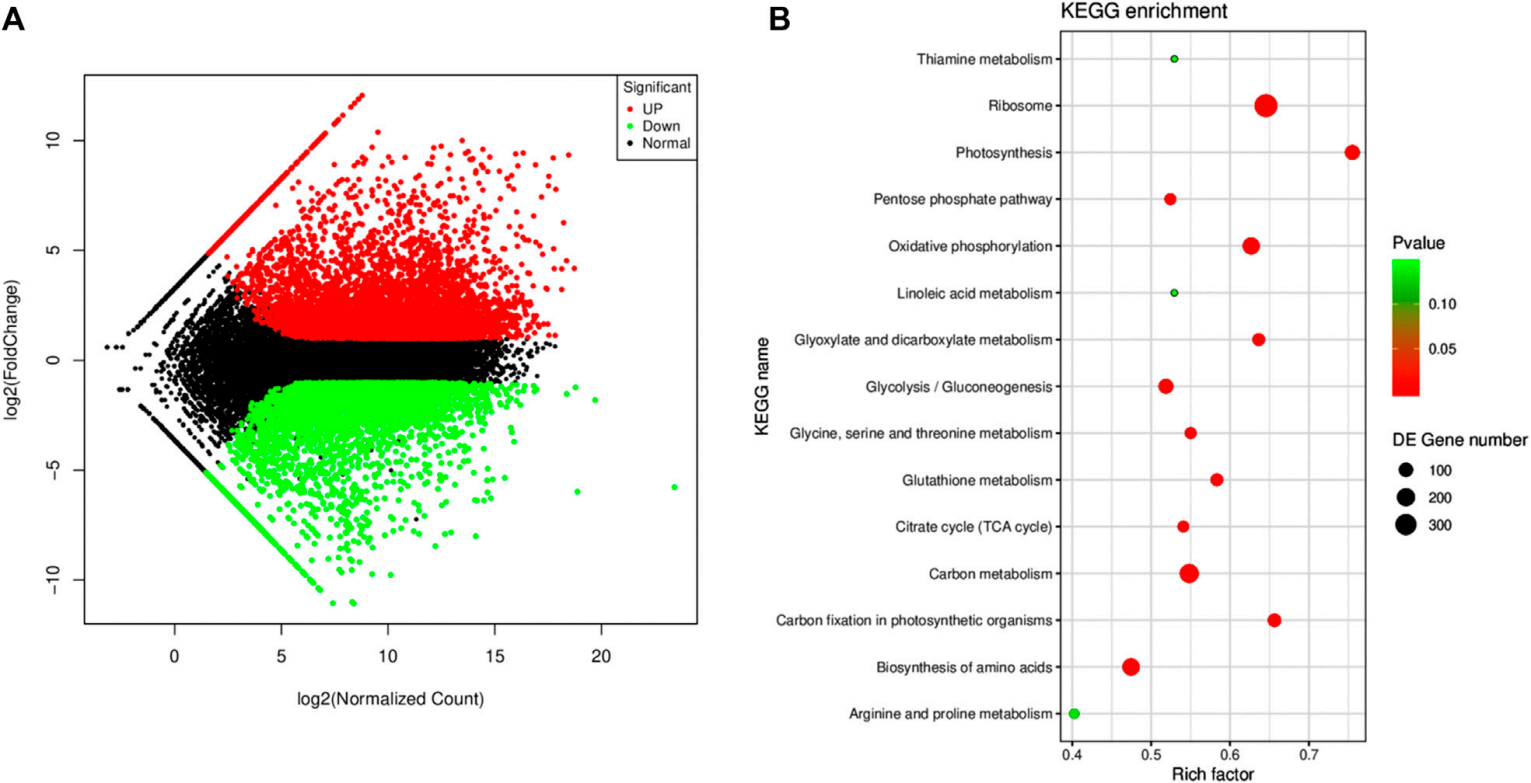
**(A)** MA map of the differentially expressed genes (*n* = 3) in Manchurian wild rice before (CK) and after *F. asiaticum* (FA) infection. **(B)** Scatter plot of the pathways to which the DEGs were significantly enriched in CK vs. FA.

The screening criteria, i.e., log2 FC ≤ −1 and ≥1 and padj < 0.05 resulted in the identification of 11,280 DEGs; 5,933 and 5,347 were up- and downregulated, respectively (Figure 4A). The DEGs were enriched in 94 KEGG pathways. Most importantly, the DEGs were significantly enriched in the ribosome, oxidative phosphorylation, carbon metabolism, photosynthesis, carbon fixation in photosynthetic organisms, amino acid biosynthesis, glycolysis, glycine, serine and threonine metabolism, citrate cycle, tryptophan metabolism, linoleic acid metabolism, and starch and sucrose metabolism (Figure 4B).

#### 3.3.2 Expression changes in tryptophan biosynthesis and metabolism-related genes are consistent with the metabolome profiles of CK vs FA

**FIGURE 5:**
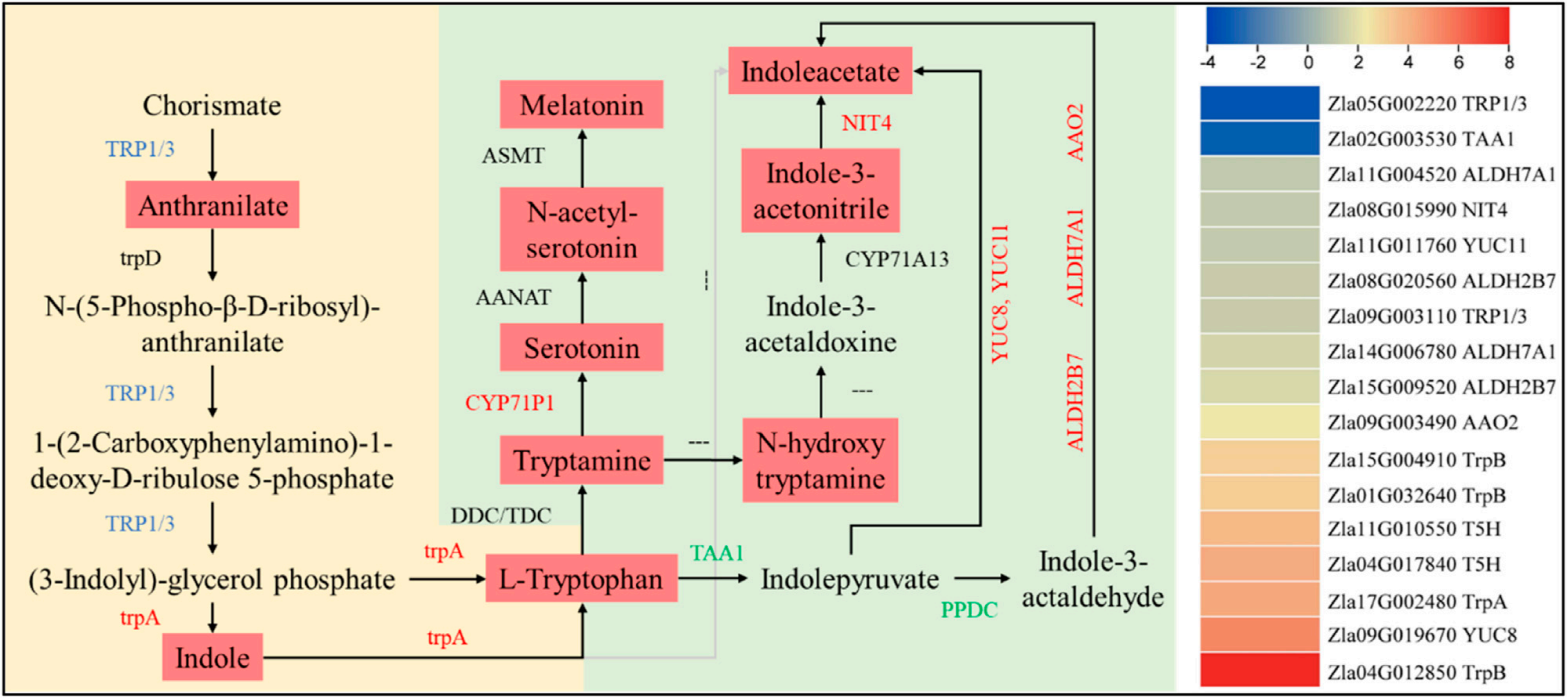
Differential regulation of tryptophan biosynthesis/metabolism pathways in CK vs. FA. The genes names in red, green, and blue represent upregulation, downregulation, and mixed regulation, respectively. The reactions with “---” indicate that the gene/enzyme is not known. The metabolites in light red boxes were highly accumulated in FA compared to CK. The heatmap on the right is based on the log2 FC of the DEGs (*n* = 3). The yellow and green backgrounds represent tryptophan biosynthesis and metabolism pathways, respectively.

A higher accumulation of tryptophan, melatonin, and indole acetate was observed in FA. In this regard, we found 17 DEGs enriched in tryptophan biosynthesis/metabolism expressed in CK vs FA. An indole-3-glycerol phosphate synthase/indole synthase (TRP1/3, *Zla09G003110*) gene was upregulated, which is consistent with the higher anthranilate accumulation in FA. It controls the conversion of chorismate to anthranilate, which is then converted to (3-indoyl)-glycerolphosphate (I3GP). The I3GP is converted to indole and then to L-tryptophan by the action of tryptophan synthase alpha chain (trpA, *Zla17G002480*). The upregulation of both trpA and TRP1/3 could be a reason for the higher tryptophan biosynthesis in FA than in CK. In contrast, the increased (or exclusive) expression of aldehyde dehydrogenases (ALDHs 7A1 [*Zla14G006780* and *Zla11G004520*) and 2B7 (*Zla15G009520* and *Zla08G020560*)], a probable aldehyde oxidase 2 (AAO2, *Zla09G003490*), Indole-3-pyruvate monooxygenase YUCCA8 and probable YUCCA 1 (YUCCA8, *Zla09G019670,* and YUCCA1, *Zla11G011760*), and a bifunctional nitrilase/nitrile hydratase NIT4 (*Zla08G015990*) in FA are consistent with IAA levels (Supplementary Tables S4, S5). The higher levels of serotonin and melatonin in FA are consistent with increased Trp and tryptamine biosynthesis, upregulation of related genes, and higher expression of tryptamine 5-hydroxylase (CYP71P1, *Zla04G017840,* and *Zla11G010550*) (Figure 5).

#### 3.3.3 Expression changes in the biosynthesis of amino acid pathway-related genes are consistent with the metabolome profiles of CK vs FA

Since the accumulation of all metabolites classified as amino acids and derivatives was increased in FA compared to CK, we checked the expression change in related genes. In total, 173 genes were enriched in the amino acid biosynthesis pathway. Among them, only 144 and 29 were up- and downregulated in FA compared to CK, respectively. The upregulated genes belonged to serine and threonine metabolism, branched-chain AA metabolism (valine, isoleucine, and leucine biosynthesis), arginine and proline metabolism, histidine metabolism, and aromatic amino acid metabolism modules of the amino acid biosynthesis (map01230; https://www.genome.jp/entry/map01230) (Supplementary Table S4).

#### 3.3.4 Expression changes in IAA and ABA biosynthesis are consistent with metabolome profiling results

Since the metabolome profile showed that both the ABA and IAA were present, we checked for the genes involved in their biosynthesis and signaling. Eleven transcripts (annotated as six genes) related to IAA biosynthesis were differentially expressed in CK vs. FA. One YUCCA8 (*Zla09G019670*) was specifically expressed in FA. In contrast, the YUCCA1 transcripts showed variable expression patterns. The AAO2 and five GH3 transcripts were upregulated in FA vs. CK. One *TAA1* gene was downregulated in FA compared to CK. The FA-specific YUCCA8 expression indicates that tryptamine is converted to N-hydroxy-TAM and then to IAA, which could be a reason for higher IAA biosynthesis in FA compared to CK. Furthermore, the higher expression of AUX1 (*Zla13G006230*), GH3s, eight of the fourteen SUARs, and downregulations of most of the IAA transcripts are consistent with the higher IAA content in FA than in CK. Thus, FA infection induces increased IAA biosynthesis and signal transduction in MWR. However, the downregulation of the ARFs and seven GH3s was also observed in FA. This indicates that MWR may experience slight variations in IAA content after FA infection (Supplementary Table S4).

**FIGURE 6:**
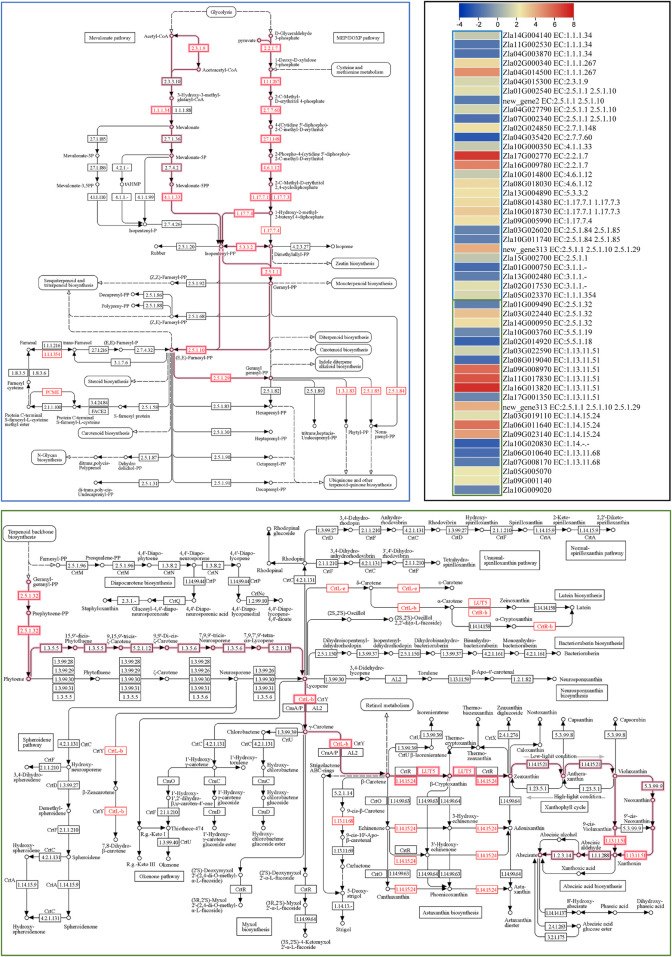
Differential regulation of terpenoid backbone (blue outline) and carotenoid biosynthesis (green outline) pathways. The highlighted pathways’ steps lead to Abscisic acid biosynthesis. Genes presented as red text were differentially expressed. The heatmap represents the log2 FC values of the differentially expressed genes (*n* = 3). The pathway maps were prepared by the KEGG pathway database (Arakawa et al., 2005).

ABA biosynthesis is a part of the carotenoid biosynthesis pathway. Therefore, we searched for “carotenoid” in the annotation file and found 25 DEGs. Geranylgeranyl pyrophosphate synthase (GGPPS), phytoene synthase PSY (PSY2 and PSY3), 9-cis-epoxycarotenoid dioxygenase NCED (NCED5, NCED3, and NCED4) and beta-carotene-3-hydroxylase 2 (crtZ2) were upregulated in FA compared to CK. On the contrary, the expression of NCED1 and one NCED4 transcript, PSY1, lycopene β-cyclase (LCB), lycopene epsilon cyclase (LcyE), and two carotenoid cleavage dioxygenase 7 (CCD7) were downregulated in FA compared to CK. The results also showed upregulation of two (SDR3a and SDR5). The SDRs convert xanthoxin to abscisic aldehyde, which is then converted to ABA (Cheng et al., 2002). These results suggest that the expression changes in GGPPS, PSY2, PSY3, NCED3, NCED5, and crtZ2 may contribute to higher ABA biosynthesis in FA since these are the major genes in the early biosynthetic steps of the carotenoid biosynthetic pathway leading to ABA biosynthesis (Chen et al., 2020). Since the terpenoid biosynthetic pathway is upstream of carotenoid biosynthesis (Figure 6), and DEGs (29) were significantly enriched in it, we also checked whether the expression changes were related to ABA biosynthesis. Notably, several genes related to C5 isoprenoid biosynthesis (both mevalonate and non-mevalonate pathways) were upregulated (Figure 6) leading to the biosynthesis of geranyl diphosphate (starting point of ABA biosynthesis in the carotenoid biosynthetic pathway). Thus, the expression changes in the terpenoid backbone and carotenoid biosynthesis pathways (beta carotene and ABA biosynthesis reactions) are consistent with ABA levels in CK vs. FA.

The higher ABA in FA triggered signal transduction, as indicated by the higher expression of ABA receptors PYL and SnRK2 transcripts. However, we also observed the upregulation of the PP2Cs in FA compared to CK, but the increase in expression was relatively lower than that of PYL and SnRK2s (Supplementary Table S4). Taken together, our results indicated that FA infection induced ABA biosynthesis and signaling in MWR.

#### 3.3.5 FA infection induces ROS generation and homeostasis in MWR

**FIGURE 7:**
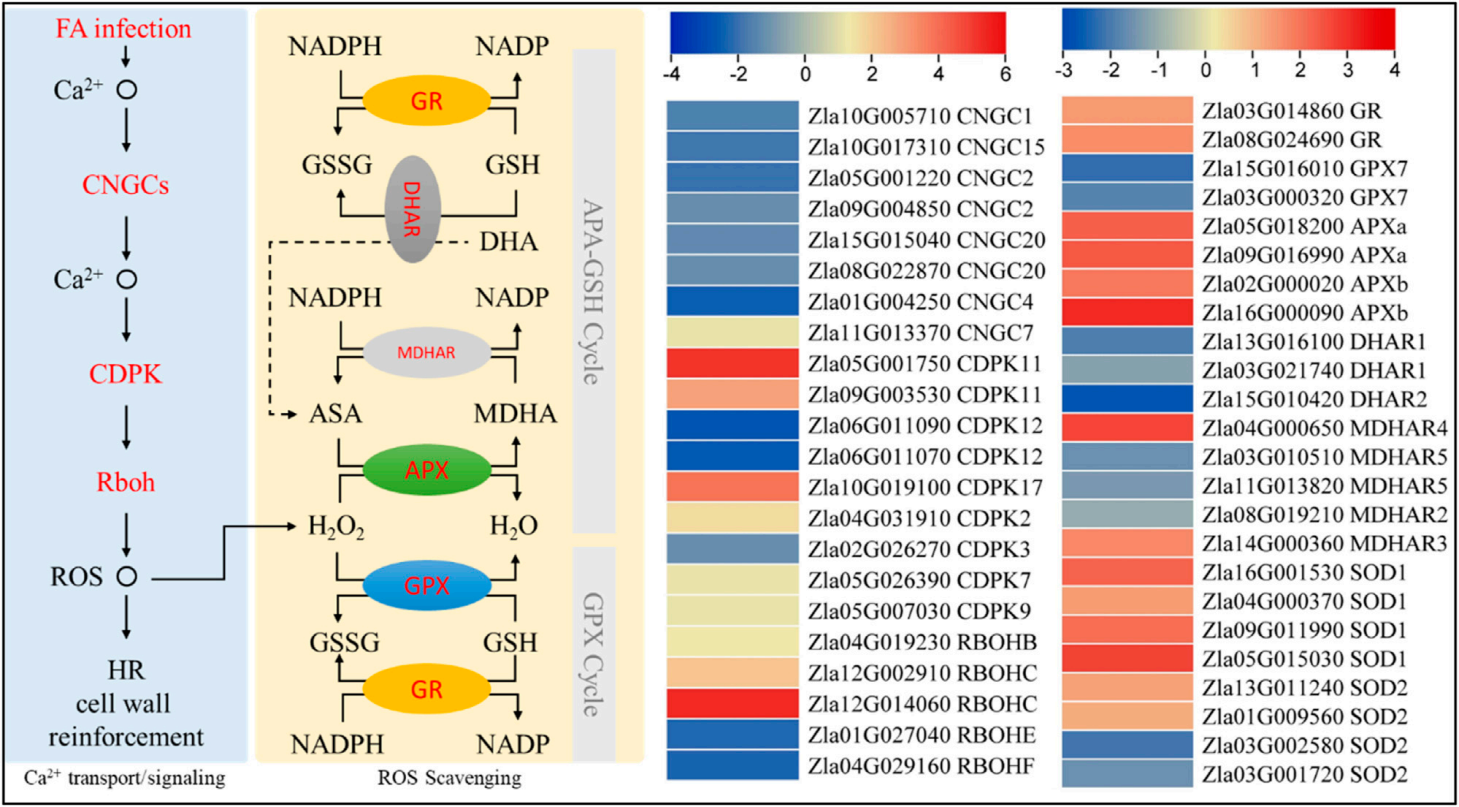
Differential regulation of reactive oxygen species generation and homeostasis in MWR infected with FA compared to CK. The genes highlighted in red text were differentially expressed. The heatmaps are log2 FC values of the differentially expressed genes (*n* = 3). CNGC, Cyclic nucleotide-gated ion channel; CDPK, calcium-dependent protein kinase; Rboh, respiratory burst oxidase homolog protein; ROS, reactive oxygen scavenging; HR, hypersensitive response; GR, glutathione reductase; GPX, glutathione peroxidase; APX, L-ascorbate peroxidase; MDHAR, monodehydroascorbate reductase; DHAR, probable glutathione S-transferase/dehydroascorbate reductase; NADP, nicotinamide adenine dinucleotide phosphate; NADPH, reduced NADP; GSSH oxidized glutathione; GSH, reduced glutathione; ASA, ascorbic acid; MDHA monodehydroascorbate; and DHA, dehydroascorbate.

Comparative transcriptome analysis revealed that a putative cyclic gated-ion channel 7 (CNGC7) and calcium-dependent protein kinases (CDPK2, 7, 9, 11, and 17) were upregulated, whereas, the other CNGCs (1, 2, 4, 15, and 20) and CDPKs (3 and 12) were downregulated in FA compared to CK. Burst oxidase homolog proteins (Rboh) B and C were upregulated, whereas Rboh E and F were downregulated in FA compared to CK. The upregulation of CNGC7, RbohB, and RbohC suggests calcium-dependent superoxide (O_2_^−^) generation. However, the downregulation of the other CNGCs and Rbohs indicates reduced ROS generation, which can be an indication of ROS homeostasis. Superoxide is converted to H_2_O_2_ through the action of superoxide dismutase (SOD). Interestingly, four SODs (SOD1 and SOD2) were upregulated in response to FA, whereas two SOD2 transcripts were downregulated in FA compared to CK. The generated H_2_O_2_ is then converted to H_2_O by the action of L-ascorbate peroxidase (APX). We observed that four APXs (two APX1 and two APX2) were upregulated in FA compared to CK. Two GPX7 transcripts showed lower expressions in FA compared to CK indicating the possibility that the GPX cycle of ROS scavenging is not active in MWR under the conditions studied. This is consistent with the reduced expressions of probable glutathione S-transferase DHARs (1 and 2). On the other hand, the upregulation of monodehydroascorbate reductase (MDHAR 3 and 4) indicates the active conversion of monodehydro ascorbate (MDA) to ascorbic acid (ASA). However, the MDHAR2 and 5 showed opposite expression trends. Finally, two glutathione reductases (GRs) showed increased expression in FA, indicating increased conversion of reduced glutathione (GSH) to oxidized glutathione (GSSG) and NADPH to NADP (Figure 7). This is consistent with the increased accumulation of GSSG in FA compared to CK (Supplementary Table S3). These expression trends indicate that ROS generation and homeostasis are active in FA-infected MWR.

#### 3.3.6 Expression changes in plant-pathogen interaction pathway

Considering the important role of the plant-pathogen interaction pathway in host responses to invading pathogens, we searched for the expression changes of genes enriched in this pathway. There were 75 differentially expressed transcripts (annotated as 15 genes); 52 and 23 were up- and downregulated, respectively, in FA compared to CK (Supplementary Table S4). All the calmodulin (CaM), heat-shock proteins 81 (htpG/HSP81), chitin elicitor receptor kinase 1 (CERK1), Pto-interacting protein 1 (PTI1), pathogenesis-related protein 1 (PR1), pathogenesis-related protein PRB1-2, and probable disease resistance protein RPS2 genes, and WRKY33 TFs were upregulated in FA compared to CK (Supplementary Table S4). On the other hand, cyclic nucleotide-gated ion channel (CNGF), nitric oxide synthase (NOA1), PTI1-like tyrosine-protein kinase 2 (PTI1-2), and mitogen-activated protein kinase kinase kinase 1 (MAPKKK1) genes were downregulated in FA compared to CK. Other genes such as calcium-dependent protein kinases (CDPKs), respiratory burst oxidase (Rboh), calcium-binding proteins (CML), disease resistance protein RPM, and RPM1-interacting proteins (RIN) showed differential expression (Supplementary Table S5). The differential expressions of CNGFs, CDPKs, Rbohs, CaM/CMLs, and NOA1 indicated that FA infection in MWR initiated a calcium signaling cascade, which led to the generation of ROS and nitric oxide (NO), resulting in a hypersensitive response (HR), cell wall changes, and possibly stomatal closure. In addition, the upregulation of WRKYs and PR1s indicates that FA infection in MWR induces defense-related gene expression (Matić et al., 2016). This is consistent with the expression changes in ROS generation and signaling-related genes, as shown in the above section (Figure 7). The activation of HR was also evident from the expression changes in RPM, RIN, RPS, and HSP81 genes.

#### 3.3.7 Expression changes in cell-wall-related genes

Cell wall modification is a dynamic barrier against pathogen invasion and in the event of a pathogen attack, multiple pathways such as plant-pathogen interaction, phytohormone biosynthesis, and signaling, and the MAPK signaling cascade, leading to cell wall modifications (Underwood, 2012). Cell wall biosynthesis, loosening, reassembly, and degradation are under the control of a broad category of genes belonging to different gene families (Nawaz et al., 2017). We searched for the DEGs related to cell wall-related DEGs and found 484 genes with at least one cell wall-related GO term (Supplementary Table S5), indicating large-scale changes in MWR after FA infection. In particular, cellulose synthase (CESA) and CESA-like (CSLs), fasciclin-like arabinogalactan proteins (FLAs), and xyloglucan glycosyltransferases (CSLCs, belonging to glycosyltransferase family 8) were downregulated in FA compared with CK. Among them, the downregulation of CESA and CSLs clearly indicates that cell wall biosynthesis is affected by FA infection. However, MWR responds to the possible effects on cellulose biosynthesis by activating/up-regulating other cell-wall-modification-related genes such as annexins, cellulases, chitinase class 1, GDSL estrases, beta-D-xylosidases, endoglucanases, glucan endo-1,3-beta-glucosidases, pectin acetylestrases, xyloglucan fucosyltransferase, pectinesterases, expansins, and peroxidases. We also observed that cell wall localized proteins, e.g., germin-like protein 1-1 [which are considered as pathogenesis-related proteins (Pei et al., 2019)], were upregulated in FA compared to CK (Supplementary Table S5). Overall, these results are consistent with the expression changes in plant-pathogen interaction and hormone biosynthesis, and signaling-related genes. The presence of possible IAA, ABA, and cell wall modifications should be further investigated based on the preliminary results presented here.

#### 3.3.8 Expression changes in carbohydrate metabolism pathways related genes

The DEGs in CK vs. FA were significantly enriched in carbohydrate metabolism [citrate cycle (53), glycolysis/gluconeogenesis (111), and starch and sucrose metabolism (94)]. Interestingly, except for five transcripts, all others enriched in the citrate cycle were upregulated in FA compared to CK, indicating that FA infection increased the oxidation and carbohydrates and fatty acids. Similarly, the genes enriched in glycolysis/gluconeogenesis were also upregulated in FA. This upregulation may also be a possible reason for increased ABA biosynthesis, as glycolysis/gluconeogenesis pathways are upstream of the terpenoid backbone and carotenoid biosynthesis pathways. While the increased expression of the genes enriched in starch and sucrose metabolism, e.g., beta-amylase, glucose-6-phosphate isomerase, alpha-amylase, alpha-glucosidase, beta-glucosidase, starch synthase, trehalose 6-phosphate synthetase, fructokinase, beta-fructofuranosidase, beta-glucosidase, hexokinase, and sucrose synthase (Supplementary Table S4) are consistent with the higher accumulation of saccharides and alcohols in FA than in CK (Supplementary Table S4). Together with the metabolomic profiles, the expression changes in pathways related to carbohydrate metabolism indicate that FA infection induces the accumulation of saccharides and alcohols in MWR.

#### 3.3.9 Expression changes in transcription factors

Transcriptome sequencing resulted in the identification of 16,944 transcription factors, of which 5,337 were differentially expressed between CK and FA (Supplementary Table S6). The differentially expressed TFs (DETs) were classified into 56 TF families. The highest number of DETs belonged to bHLH (576) followed by NAC (442), EFF (399), MYB related (360), and WRKY (298) (Supplementary Table S6). Overall, we observed that a relatively higher number of TFs were upregulated in FA compared to CK, with the exception of HRT-like, HB-PHD, ARR-B, ZF-HD, GATA, ARF, HD-ZIP, LBD, M-type MADS, and C3H family members. The most upregulated TFs in FA were bHLH, WRKY, NF-YB, C2H2, NAC, bZIP, and AP2. Meanwhile, the most downregulated TFs in FA were DBB, trihelix, bHLH, E2F/DP, G2-like, LBD, and GATA. These results indicate that FA infection in MWR induces large-scale transcriptional changes in MWR.

### 3.4 qRT-PCR analyses

**FIGURE 8:**
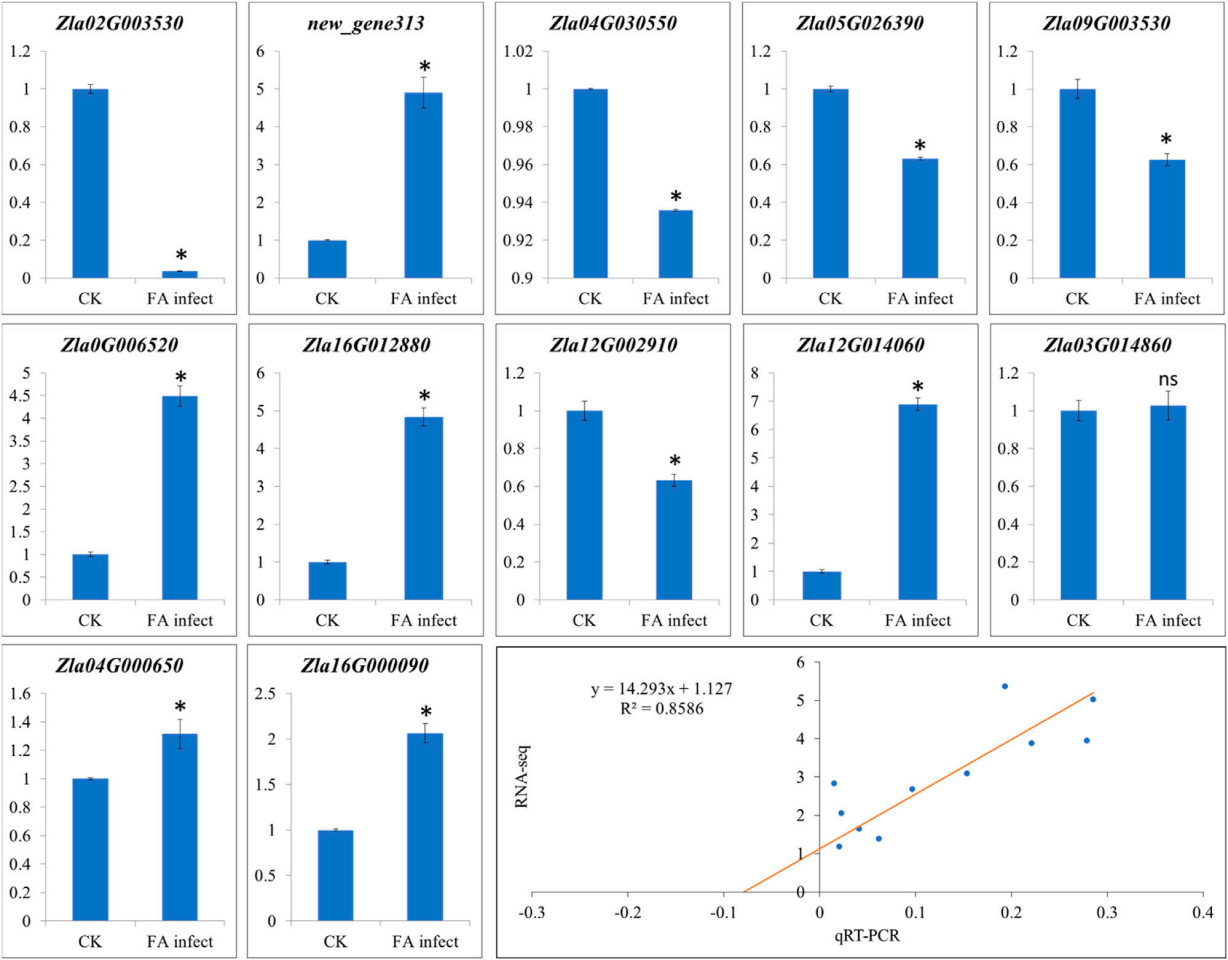
Quantitative real-time PCR analysis of WMR genes in FA infect (infected with *F. asiaticum*) and CK (control). The x-axis and y-axis represent treatments and relative gene expression, respectively. The bars show mean ± SD of *n* = 3. Where CK and FA infect show control (no *F. asiaticum* infection) and MWR infected with *F. asiaticum.* * and ns show that the means differ significantly and non-significantly, respectively at *p* < 0.05. The last figure panel shows the correlation between RNA-seq and qRT-PCR data.

The qRT-PCR analyses of twelve genes (Supplementary Table S1) were performed to confirm the expression observed in the RNA-seq analysis. The qRT-PCR analysis showed that the selected genes showed similar expression trends as of RNA-seq results (Figure 8) with correlation coefficient of 0.8586 (Figure 8), thus validating the RNA-seq results.

## 4 Discussion

Human selection of crops during domestication has led to the alteration of and even reductions in the diversity of secondary metabolite content in crops, including rice. Such changes occur directly and indirectly through the selection pressure on the genes that control the production of secondary metabolites (Ku et al., 2020; Alseekh et al., 2021; Chen et al., 2021; Ben-Abu and Itsko, 2022). This has made most commercially important crops more susceptible to a range of abiotic and biotic stresses. For example, all high-yielding international rice varieties are highly susceptible to sheath rot disease (Jamaloddin et al., 2021), possibly as a result of changes during rice domestication or human breeding efforts. Recently, efforts have been made to deploy wild relatives for genetic improvement of rice (*O. sativa*) to achieve food and nutritional security for humankind (McCouch et al., 2007; Ehsan and Georgia, 2014; Brar and Khush, 2018; Gaikwad et al., 2021). Keeping this in mind, the present study applied integrated metabolomic and transcriptomic profiling of MWR under CK and FA to gain insights into the sheath rot resistance.

### 4.1 MWR induces melatonin and amino acids biosynthesis in response to FA infection

Melatonin is known to be involved in the regulation of tolerance to various plant diseases. For example, melatonin enhances disease resistance to *Botrytis cinerea* in tomato fruit by altering jasmonic acid signaling and H_2_O_2_ levels and improves resistance to pathogens in *Arabidopsis* through NO signaling (Shi et al., 2015; Fan et al., 2018; Liu et al., 2019). Our results that melatonin levels were higher in FA than CK (fold change = 144.02) suggest that MWR attempts to mitigate the effect of FA attack (Table 1; Supplementary Table S2). Furthermore, the increased accumulation of 31 alkaloid compounds in MWR in response to FA infection (Supplementary Table S2). These results are consistent with the known protective role of alkaloids against predators, as well as improve plants’ tolerance to pathogens (Matsuura and Fett-Neto, 2017; Gao et al., 2020; Zeng et al., 2022). *Arabidopsis* serotonin N -acetyltransferase knockout mutant plants show decreased melatonin and SA levels, resulting in susceptibility to an avirulent pathogen, i.e., *Pseudomonas syringe* pv. Tomato DC3000 (*PstDC3000*) (Lee et al., 2015), which is consistent with our results. Therefore, the increase in melatonin content in response to FA attack may contribute to the tolerance of MWR to FA.

Another study showed that rice leaves infected with *Bipolaris oryzae* accumulated higher levels of serotonin, which served as a substrate for POD in the presence of H_2_O_2_, resulting in the formation of a complex mixture of oligomerics that function as a physical barrier against the spread of pathogen infection (Ishihara et al., 2008). For example, rice lesion mimic mutant, *Sekiguchi lesion* treated with serotonin showed effective suppression of the fungal growth and activate the expression of some resistance genes, such as probenazole 1 (*PBZ1*), phenylalanine ammonia-lyase 1 (*PAL1*), chitinase 1 and 3 (*Cht1* and *Cht3*), and others (Fujiwara et al., 2010). Therefore, the discovered genes and alkaloid compounds (Supplementary Tables S2, S4) are potential targets for improving tolerance against FA in cultivated rice.

Engineering of metabolic pathways in plants holds great promise for increasing the abundance of specific metabolites with functions in disease tolerance (Zorrilla-López et al., 2013; Calgaro-Kozina et al., 2020). Tryptophan is a precursor for the biosynthesis of auxin and secondary metabolites that protect plants against fungal, bacterial, and insect attack (Ishihara et al., 2008; Consonni et al., 2010; Legault et al., 2011; Miao et al., 2019; Pastorczyk et al., 2020; Kosaka et al., 2021). Thus, the expression changes of tryptophan biosynthesis genes [*TRP1/3*, *trpA*, *ALDHs 7A1*, *YUCCA8*, *YUCCA11*, *NIT4,* and *CYP71P1* (Figure 5; Supplementary Table S4)] in FA and the resulting increased tryptophan content suggest their involvement in MWR responses/tolerance to FA attack. Previous studies have demonstrated similar roles for tryptophan. For example, the increased expression of CYP71A12 (monooxygenase) triggered tryptophan metabolism in response to pathogen attack and enhanced *Arabidopsis* immunity in *Arabidopsis* by inducing indole-3-carboxylic acid biosynthesis (Pastorczyk et al., 2020). Previous studies have shown that amino acids and their metabolites enhance plant immunity to various biotic stresses (Kadotani et al., 2016). For example, isoleucine enhances *Arabidopsis* resistance to *B. cinerea* via the jasmonate signaling pathway (Li et al., 2021), and L-histidine induces resistance in tomato and *Arabidopsis* plants to the bacterial pathogens *Ralstonia olanacearum* and *B. cinerea*, respectively, in part by activating ethylene signaling (Seo et al., 2016). In addition, a study by Kadotani et al. (2016) showed that the exogenous application of the amino acid glutamate to rice induced resistance to *Magnaporthe oryzae* (a hemi-biotrophic fungus that causes blast disease) by the activating of host defenses through a mechanism based on an SA signaling pathway. The increased accumulation of 13 amino acids and derivative compounds are expression changes in genes enriched in the biosynthesis of the amino acid pathway, suggesting that amino acids play an important role in tolerance to FA in MWR (Seo et al., 2016).

### 4.2 IAA and ABA biosynthesis and signal transduction increased in MWR

Plant growth and responses to environmental signals are largely regulated by phytohormones and their signaling transduction pathways (Denancé et al., 2013). Phytohormone signaling pathways are known to be targeted by pathogens to disrupt and evade plant defense mechanisms (Denancé et al., 2013; Ma and Ma, 2016) in order to colonize plant tissues. Pathogens can also hijack plant developmental and nutrient allocation processes modulated by “growth” hormones to facilitate sustained colonization and dissemination (Ma and Ma, 2016). Auxins (e.g., IAA) have been reported to regulate developmental processes such as cell wall architecture, root morphology, and stomatal development/function (Denancé et al., 2013). Since tryptophan is the common precursor for both melatonin and IAA (Zhang et al., 2022b), these two compounds therefore act synergistically to regulate plant growth and stress resistance (for details see Zhang et al. (2022b)). The observation that IAA (kz000278) accumulation was increased by ∼5-fold in FA compared to CK is consistent with these reports (Supplementary Table S2). Furthermore, it has been documented that conjugated Aux-aspartic acid (IAA-Asp) plays a key role in modulating resistance to the necrotrophic fungus *B. cinerea* and *PstDC3000* (Denancé et al., 2013). For example, *Arabidopsis*, tomato, and *Nicotiana benthamiana* infected with the two pathogens (*B. cinerea* and *PstDC3000*) increased the expression of *GH3.2* and *GH3.4* genes, which encode two enzymes required for conjugation of Aux (IAA) with Asp (Denancé et al., 2013). The higher (∼2.59-fold) expression of the IAA gene (*GH3.8, Zla05G012430*) upon FA attack compared to CK (Supplementary Table S4), indicates its possible involvement in regulating the MWR response to FA. The Aux-related DAMs and DEGs related to Aux could be useful for functional validation experiments to deepen our understanding of their roles in modulating MWR response to FA (Wang et al., 2022).

ABA is an essential signal for plant resistance to pathogens that affect JA or SA biosynthesis and the activation of plant defenses (Adie et al., 2007; Fan et al., 2009; Denancé et al., 2013; Xu et al., 2013). It can act as a repressor or enhancer of plant defense depending on the type of biotic stress (Denancé et al., 2013). For example, ABA-impaired (biosynthesis or signaling) mutants in tomato and several other *Arabidopsis* mutants (*abi1-1, abi2-1, aba1-6, aba2-12, aba3-2,* and *pyrlpylpyl2pyl4*) showed overexpression of defense signaling pathway genes. These mutants showed enhanced resistance to various pathogens, i.e., *B. cinerea, F. oxysporum*, *P. syringae*, *Plectoshpaerella cucumerina* and *Hyaloperonospora parasitica* as summarized in Denancé et al. (2013). On the other hand, ABA promoted susceptibility to the rice leaf blight pathogen *Xanthomonas oryzae* pv *oryzae* by suppressing SA-mediated defenses (Xu et al., 2013). The increased accumulation of ABA (and associated -metabolites) and consistent expression changes in related genes suggest that ABA plays an important role in MWR against FA attack. Future functional validation studies should be conducted out to understand how these genes and ABA modulate resistance/tolerance in MWR to FA attack (Figure 6; Supplementary Tables S2, S4).

The expression changes in a large number of IAA and ABA biosynthesis and signaling-related genes suggest that these hormones play a central role in regulating MWR to FA attack, which is consistent with a previous report on rice infected by *S. oryzae and P. fuscovaginae* (Peeters et al., 2020). Also, the reduced expression of carotenoid biosynthesis-related genes such as *NCED1*, *PSY1, LCB, LcyE,* and *CCD7* upon FA attack in MWR (Supplementary Table S4) is consistent with Boba et al. (2011), these genes could have either caused the diversion of substrates to produce other constituents of isoprenoid pathway or somehow activated other branches of this pathway (Boba et al., 2011). Carotenoids are known to directly scavenge ROS in biological membranes, thereby protecting them from damage caused by various stresses including FA (Strzałka et al., 2003; Kim et al., 2018).

### 4.3 Hypersensitive response (HR) is activated in MWR after FA infection

Plants use various immune responses to protect themselves from pathogen attack, one of which is hypersensitive response (HR) (Fujiwara et al., 2010). Hypersensitive responses are of two types, i.e., HR achieved by programmed cell death and HR induced coordinately induced with production of ROS, lignification, biosynthesis of phytoalexins, and expression of defense-related genes. Our results that the expression of SODs, APXs, GRs, and POD transcripts were increased in FA compared to CK (Figure 7; Supplementary Table S4) indicate that an antioxidant defense mechanism is activated in MWR to reduce/mitigate the effect of FA attack. These expressions also suggest that MWR perceives the pathogen (FA) in order to activate defense-related genes such as CaM, htpG/HSP81, CERK1, PTI1, PR1, PRB1-2, and RPS2 (Figure 7; Supplementary Tables S4, S5) (Lehmann et al., 2015). These observations are consistent with previous reports that several PR genes are upregulated in response to *Fusarium* infection in *Allium sativum* (Anisimova et al., 2021). Transcriptome analysis of alfalfa plants infected with *Fusarium* (root rot) also showed similar expression trends of PTI1, PR proteins, and CaM genes (Zhang et al., 2022c). Therefore, our results suggest that MWR uses antioxidant defense genes for ROS homeostasis when infected with FA.

ROS has been implicated in the HR via cell wall reinforcement at the site of infection (Lehmann et al., 2015). The transcriptome data indicate that MWR also modifies cell walls in response to FA attacks (Supplementary Table S4) (Underwood, 2012; Nawaz et al., 2017). Xyloglucan has been characterized as a potent elicitor associated with cell wall-associated immunity in plants (Choi et al., 2013; Molina et al., 2021; Wan et al., 2021). Exogenous application of xyloglucan oligosaccharides accelerated cell elongation, and division along with the expression of defense-responsive genes, including the jasmonate ZIM domain gene *JAZ8* and chitinase-like gene *ATHCHIB* in tobacco (González-Pérez et al., 2014). The expression changes in annexins, cellulases, chitinase class 1, GDSL estrases, beta-D-xylosidases, endoglucanases, glucan endo-1,3-beta-glucosidases, pectin acetylestrases, xyloglucan fucosyltransferase, pectinesterases, and expansins indicate that MWR cell walls undergo large-scale changes when infected with FA. These observations have been reported in different plant species against a range of pathogens (Malinovsky et al., 2014). The HR strategies in plants are also linked with IAA and melatonin (Zhang et al., 2022b). IAA leads to swelling of the cell wall (Majda and Robert, 2018), while melatonin may induce changes in the expression changes of *SAUR* family members to reduce cell wall expansion and limit the potential pathogen invasion. Taken together, our results indicate that MWR activates HR in response to FA attack.

Many plant responses to fungal attack are closely linked to the pathways that modulate the levels of sugar/carbohydrate in the plant cell and maintain energy homeostasis (Hey et al., 2010). Carbohydrates play an important role by acting as signaling molecules through interaction with the hormonal signaling network. They also regulate the plant immune system and cellular activity at various levels, from transcription and translation to protein stability and activity (Rolland et al., 2006). Many genes involved in carbohydrate metabolism such as hexokinases (HXKs), were upregulated in MWR upon FA infection (Supplementary Table S4). According to Smeekens et al. (2010), HXKs are ideal (enzymatic) glucose sensors that catalyze the first step of glycolysis (i.e., the conversion of glucose to glucose6-phosphate). HXK isoforms are found in the cytosol, chloroplasts, mitochondria, and the nucleus (Morkunas and Ratajczak, 2014). Specifically, mitochondria-associated HXKs have been demonstrated to regulate programmed cell death and promote the expression of many of the PR genes induced during programmed cell death (PCD) (Kim et al., 2006), suggesting that some of features of HR cell death are conserved in the HXK-mediated PCD process.

## 5 Conclusion

We explored comparative metabolome and transcriptome profiles of an MWR cultivar Zhejiao NO.7 before and after infection with *F. asiaticum*, which is commonly known as sheath rot disease. Our preliminary results have shown that the cultivar studied exhibits tolerance symptoms. The global metabolome profile metabolites related to 29 compound classes are differentially accumulated in response to *F. asiaticum* infection. Most prominent results suggest that most compound classes are up-accumulated in response to infection. The up-accumulated metabolites were enriched in tryptophan metabolism, phenylalanine, tyrosine, and tryptophan biosynthesis, isoflavonoid biosynthesis, flavonoid biosynthesis, indole alkaloid biosynthesis, biosynthesis of amino acids, and anthocyanin biosynthesis, therefore, we explored more details about these pathways. The transcriptome profiling showed that the genes enriched in the above-mentioned pathways showed expression profiles that are consistent with metabolite accumulation of the same pathways. Thus, we conclude that tryptophan biosynthesis and metabolism, amino acid biosynthesis, plant-pathogen interaction, ROS homeostasis, cell-wall modification, and carbohydrate metabolism are key pathways through which MWR responds to *F. asiaticum* infection.

## Data Availability

The original contributions presented in the study are included in the article/Supplementary Material, further inquiries can be directed to the corresponding authors.

## Supplementary material

SUPPLEMENTARY FIGURE S1 Principal component analysis, **(B)** hierarchical cluster analysis, and **(C)** Pearson’s correlation coefficient analysis of the relative metabolite intensities in MWR before (CK) and after *F. asiaticum* (FA) infection.

SUPPLEMENTARY FIGURE S2 Volcano plot of KEGG pathway in which the differentially accumulated metabolites were significantly enriched in MWR before (CK) and after *F. asiaticum* (FA) infection.
