# Supplementary figures and images for: Integrated transcriptome and metabolome analysis unveil the response mechanism in wild rice (Zizania latifolia griseb.) against sheath rot infection
Source: Front Genet. 2023 Jun 9;14:1163464. doi: 10.3389/fgene.2023.1163464 (PMC10289006; doi:10.3389/fgene.2023.1163464)

## Slide 1
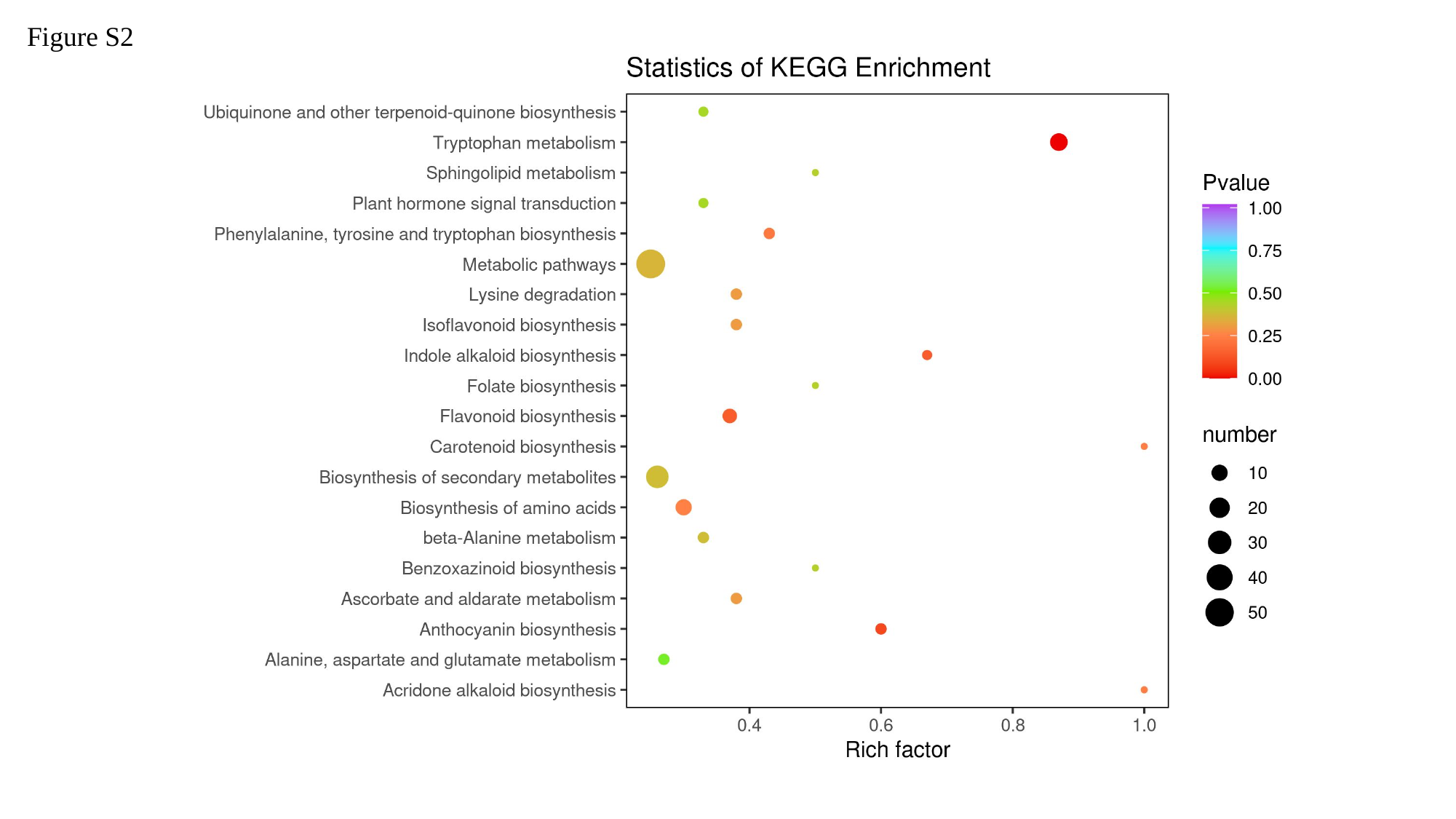

Figure S2

Supplement: Supplementary file 1 [file Presentation1.PPTX]

Figure S1A

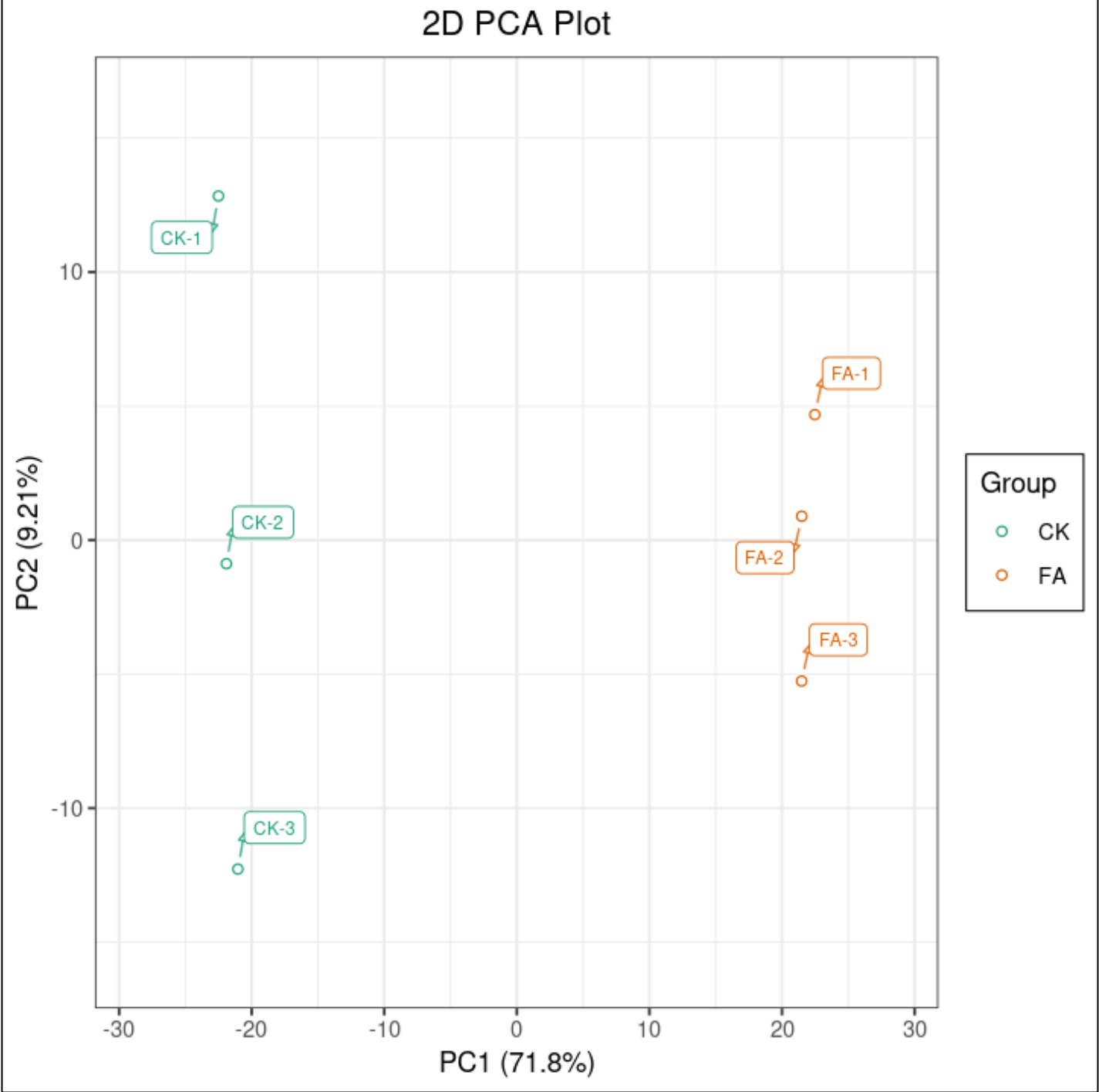

Figure S1B

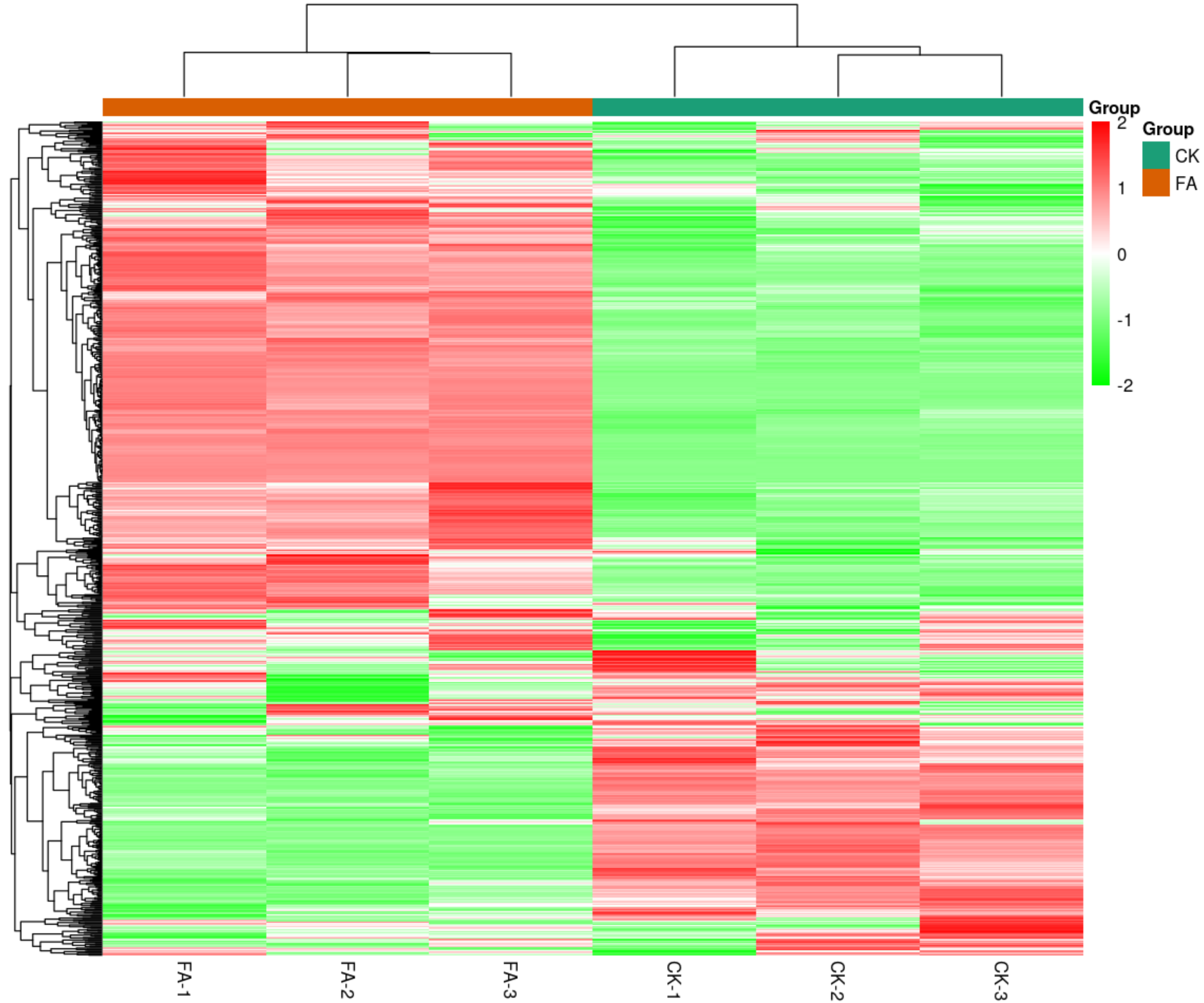

Figure S1C

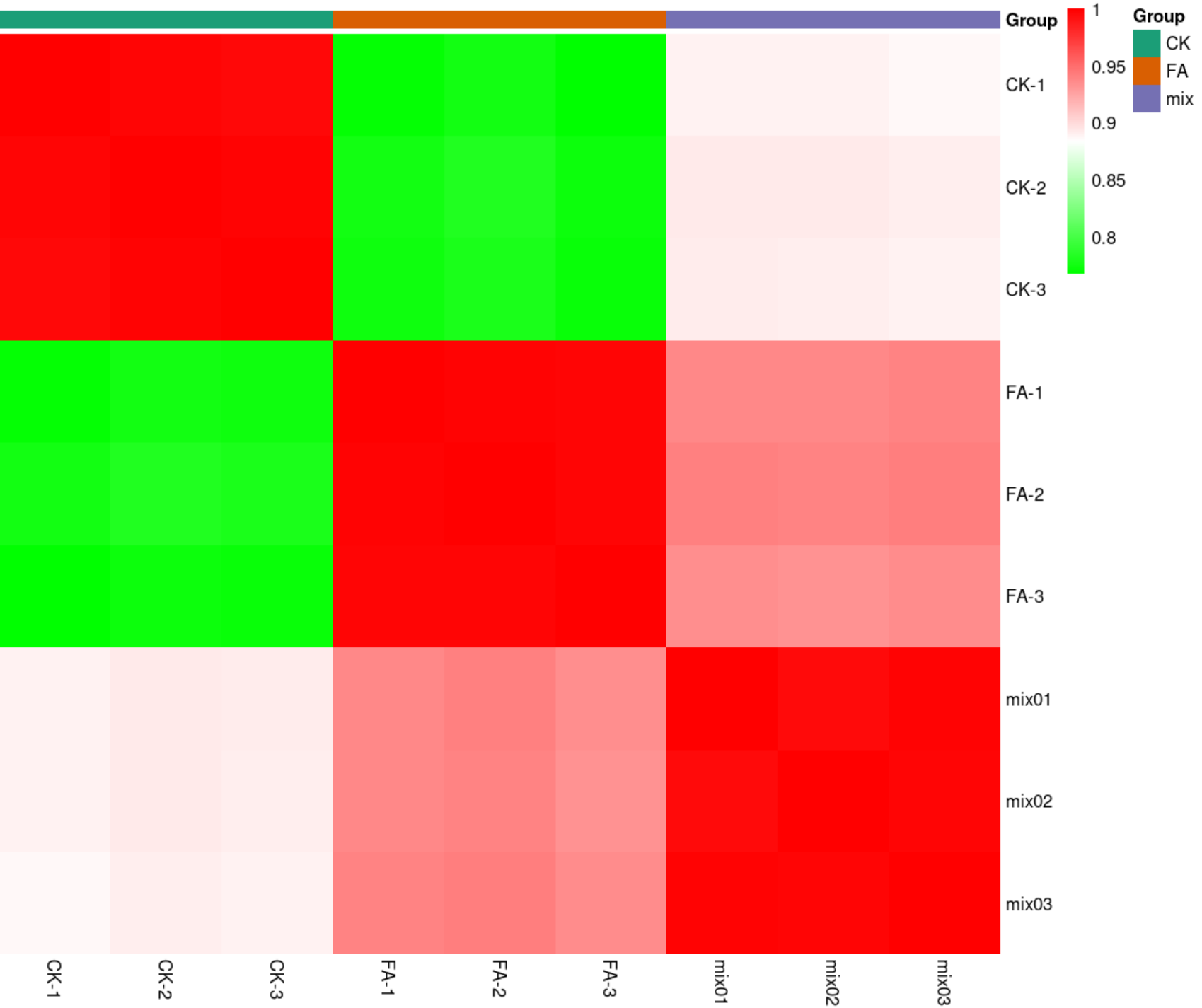

Supplement: Supplementary file 2 [file DataSheet1.PDF]
